# Epstein-Barr Virus Down-Regulates Tumor Suppressor *DOK1* Expression

**DOI:** 10.1371/journal.ppat.1004125

**Published:** 2014-05-08

**Authors:** Maha Siouda, Cecilia Frecha, Rosita Accardi, Jiping Yue, Cyrille Cuenin, Henri Gruffat, Evelyne Manet, Zdenko Herceg, Bakary S. Sylla, Massimo Tommasino

**Affiliations:** 1 International Agency for Research on Cancer, World Health Organization, Lyon, France; 2 CIRI, International Center for Infectiology Research, Oncogenic Herpesviruses Team, Université de Lyon, Lyon, France; 3 Inserm, U1111, Lyon, France; 4 Ecole Normale Supérieure de Lyon, Lyon, France; 5 Université Lyon 1, Centre International de Recherche en Infectiologie, Lyon, France; 6 CNRS, UMR5308, Lyon, France; University of North Carolina at Chapel Hill, United States of America

## Abstract

The *DOK1* tumor suppressor gene encodes an adapter protein that acts as a negative regulator of several signaling pathways. We have previously reported that *DOK1* expression is up-regulated upon cellular stress, via the transcription factor E2F1, and down-regulated in a variety of human malignancies due to aberrant hypermethylation of its promoter. Here we show that Epstein Barr virus (EBV) infection of primary human B-cells leads to the down-regulation of *DOK1* gene expression via the viral oncoprotein LMP1. LMP1 alone induces recruitment to the *DOK1* promoter of at least two independent inhibitory complexes, one containing E2F1/pRB/DNMT1 and another containing at least EZH2. These events result in tri-methylation of histone H3 at lysine 27 (H3K27me3) of the *DOK1* promoter and gene expression silencing. We also present evidence that the presence of additional EBV proteins leads to further repression of *DOK1* expression with an additional mechanism. Indeed, EBV infection of B-cells induces DNA methylation at the *DOK1* promoter region including the E2F1 responsive elements that, in turn, lose the ability to interact with E2F complexes. Treatment of EBV-infected B-cell-lines with the methyl-transferase inhibitor 5-aza-2′-deoxycytidine rescues *DOK1* expression. In summary, our data show the deregulation of *DOK1* gene expression by EBV and provide novel insights into the regulation of the *DOK1* tumor suppressor in viral-related carcinogenesis.

## Author Summary

Many oncogenic viruses exhibit cellular transforming properties, often involving oncogenes activation and tumor suppressor genes inactivation. The *DOK1* gene is a newly identified tumor suppressor gene with altered expression via hypermethylation of its promoter in a variety of human cancers, including head and neck, lung, gastric and others. In addition, a correlation has been reported between *DOK1* aberrant hypermethylation and the presence of oncogenic viruses such as hepatitis B virus (HBV) in hepatocellular carcinoma (HCC) and Epstein-Barr virus (EBV) in Burkitt's lymphoma-derived cell lines. Here we demonstrate for the first time that EBV is directly involved in the inhibition of *DOK1* expression in B-cells. We show that EBV leads to epigenetic repression of *DOK1* through increased DNA methylation of its promoter and H3K27 tri-methylation. The LMP1 oncoprotein plays a key role in the repression of *DOK1* expression. It promotes the formation and the recruitment to the *DOK1* promoter of transcriptionally inhibitory complexes composed of E2F1/pRB/DNMT1 and of EZH2 which is part of the polycomb repressive complex 2. Interestingly, one or more additional EBV protein(s) cooperate(s) with LMP1 in inducing massive DNA methylation at the *DOK1* promoter, leading to the loss of E2F1 complexes recruitment and even stronger repression of *DOK1* expression.

## Introduction

Cellular transformation induced by oncogenic viruses often involves the activation of growth-promoting signaling pathways and the inactivation of tumor suppressor genes. The downstream of tyrosine kinase 1gene (*DOK1*) has emerged as a newly identified tumor suppressor gene that encodes a multi-domain adapter protein and acts as a negative regulator of signaling pathways involved in several cellular functions. DOK1 inhibits cell proliferation, down regulates MAP kinase activity, and has an opposing role in leukemogenesis and promotes cell spreading, motility, and apoptosis [1], [2]. Functional studies showed that mice lacking the *DOK1* and/or *DOK2* genes have a high susceptibility to the development of lung adenocarcinomas [3] and exhibit significant defects in their immune responses and immune cell development, often developing myelo-proliferative and autoimmune diseases, e.g. lupus-like renal disease [4], [5]. The *DOK1* gene locus is located in the human chromosome 2p13 region, which is frequently rearranged in a number of human tumors [6]. Oncogenic tyrosine kinases such as p210^BCR-ABL^, the causative mutation in chronic myelogenous leukemia (CML), and Src target DOK1 for ubiquitin-mediated proteasomal degradation [7], therefore promoting cell proliferation. We have reported a frameshift mutation of the *DOK1* gene in chronic lymphoid leukemia (CLL) resulting in the expression of truncated DOK1 that is exclusively localized in the nucleus and loses its tumor suppressive activities, in contrast with the cytoplasmic wild type protein [8]. We also showed that *DOK1* gene expression is repressed in a large proportion of head and neck cancer (HNC), lung cancer and Burkitt's lymphoma [9], as a result of aberrant hypermethylation of its promoter region. The inactivation of *DOK1* through promoter methylation also occurred in liver and gastric cancers [10], [11]. Thus, *DOK1* emerged as a tumor suppressor frequently altered in a variety of human cancers, making it a potential marker and therapeutic target in cancer control.

Epstein-Barr virus (EBV) is a γ-herpes-virus that is widespread in 90% of human populations. In the majority of individuals, EBV persists as a permanent, asymptomatic infection of the lymphocytes B-lymphocyte pool [12]. EBV occasionally causes infectious mononucleosis in adolescents [13] and is considered a human carcinogenic infectious agent. Indeed, EBV is associated with the development of different types of B-cell lymphoma such as Burkitt's lymphoma (BL), Hodgkin disease, lympho-proliferative disorders in immuno-deficient individuals, and nasopharyngeal carcinoma [14], [15], [16]. EBV is also associated with gastric cancer [17]. The oncogenic potential of EBV has been further demonstrated by its ability to immortalize efficiently the primary human B-cells *in vitro* in lymphoblastoid cell lines (LCLs) [18]. LCLs carry the EBV genome in an extra-chromosomal episome state and express nine latent viral proteins: three trans-membrane proteins (LMP1, LMP2A and 2B) and six nuclear antigens (EBNAs 1, 2, 3A, 3B, 3C and LP), along with other non-translated RNA products [12]. These viral products enhance the proliferation of quiescent B-cells and maintain the viral genome in its episomal form. However, only EBNA1, 2, 3A, 3C, LP, and LMP1 are essential for the transformation of primary B-cells into LCLs [19]. The latent membrane protein 1 (LMP1) is crucial for EBV-induced B-cell immortalization. It is the only EBV latent protein that displays transforming properties *in vitro* [20].

LMP1 protein is thought to alter cell growth transformation by mimicking the activated forms of tumor necrosis factor receptor (TNFR), CD40 and CD30 receptors [21], [22], [23]. Through its long C-terminal cytosolic domain, LMP1 has the ability to induce several signaling pathways, including the MAP kinase (both ERK/MAPK and p38/MAPK), nuclear factor kappa B (NF-κB) and c-Jun N-terminal kinase (JNK) [24], [25], [26], [27]. The alteration of these signaling pathways by LMP1 is essential for the oncogenicity of EBV.

The presence of the EBV genome in several lymphomas, and its ability to induce B-cell immortalization, and alter host-cell expression profiles and epigenome (i.e. DNA methylation patterns) strongly support an etiological role for EBV in these cancers. We recently reported that the expression of *DOK1* gene is repressed through DNA hypermethylation in BL cell lines, it became of interest to investigate the possible role of EBV in the inhibition of *DOK1* expression in infected B-cells. To date, very little is known about the regulation of *DOK1* expression by oncogenic viruses.

In the present study, we demonstrate a strong association between EBV infection and *DOK1* gene silencing via hypermethylation of its promoter in EBV-infected cell lines. We show that EBV infection in B-cells leads to epigenetic repression and CpG methylation of the *DOK1* gene and that LMP1 expression inhibits *DOK1* promoter activity via the recruitment of inhibitory complexes including E2F1, pRB, DNMT1 and EZH2.

## Results

### EBV infection of primary human B-cells *in vitro* leads to down-regulation of *DOK1* expression

**Figure 1:**
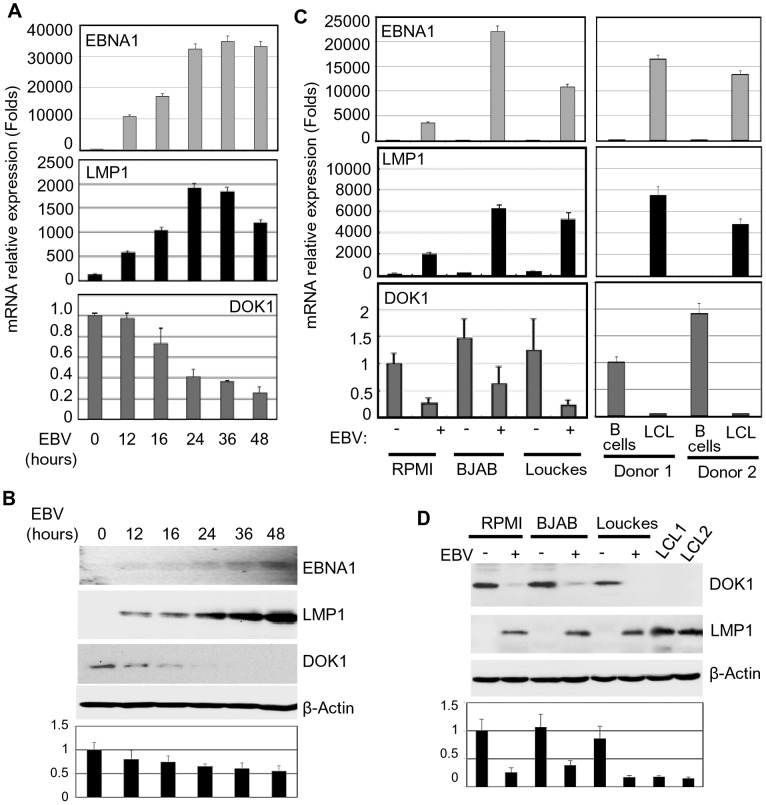
EBV infection in vitro inhibits *DOK1* gene expression. Primary B-cells were isolated from healthy donor blood using negative selection, and then infected with GFP-EBV recombinant virus. (**A**) mRNA levels of *EBNA1*, *LMP1*, and *DOK1* were measured using real time PCR at different time points 12, 16, 24, 36, and 48 hours post infection and normalized to *GAPDH* expression. The isolated primary B-cells were used as a control (time point 0). Data are average of three independent experiments. (**B**) DOK1, LMP1, EBNA1, and β-actin protein levels were determined by western blotting. (**C**) Total mRNA was extracted from RPMI, BJAB and Louckes cells two weeks after infection with GFP-EBV recombinant virus. The respective non-infected cells were used as control. Similarly, mRNA was extracted from two LCLs and their original primary B-cells. The expression levels of EBNA1, LMP1, and *DOK1* were measured by real time PCR and normalized to *GAPDH* expression. (**D**) DOK1, LMP1 and β-actin protein levels were determined by western blotting. DOK1 protein levels were quantified from two independent immunoblots and normalized to the corresponding β-actin level (bottom of B and D).

Based on our previous results that showed the down-regulation of *DOK1* expression in BL cell-lines [9], we evaluate whether this event was linked to infection with EBV, a key risk factor for this malignancy. Primary human B-cells, isolated from different healthy donors, were infected in independent experiments with recombinant EBV virus expressing the green fluorescent protein (GFP-EBV). The infection efficiency was evaluated by flow cytometry to monitor GFP expression (data not shown). The expression of EBV genes *EBNA1* and *LMP1*, as well as *DOK1* was determined by real-time PCR and western blot at different time points post-infection (**Figure 1A and B**). EBV infection resulted in a strong reduction of *DOK1* mRNA and protein levels, which was evident at 16 hours post-infection (**Figure 1A**). Similarly, *DOK1* mRNA and protein levels were strongly down-regulated by EBV in three cancers B-cell lines (RPMI, BJAB and Louckes) infected by EBV, as well as in EBV-immortalized lymphoblastoid cells lines (LCLs) (**Figure 1C and D**). Together, these findings highlight a role for EBV in down-regulating *DOK1* gene expression.

### LMP1 plays a key role in the inhibition of *DOK1* expression

**Figure 2:**
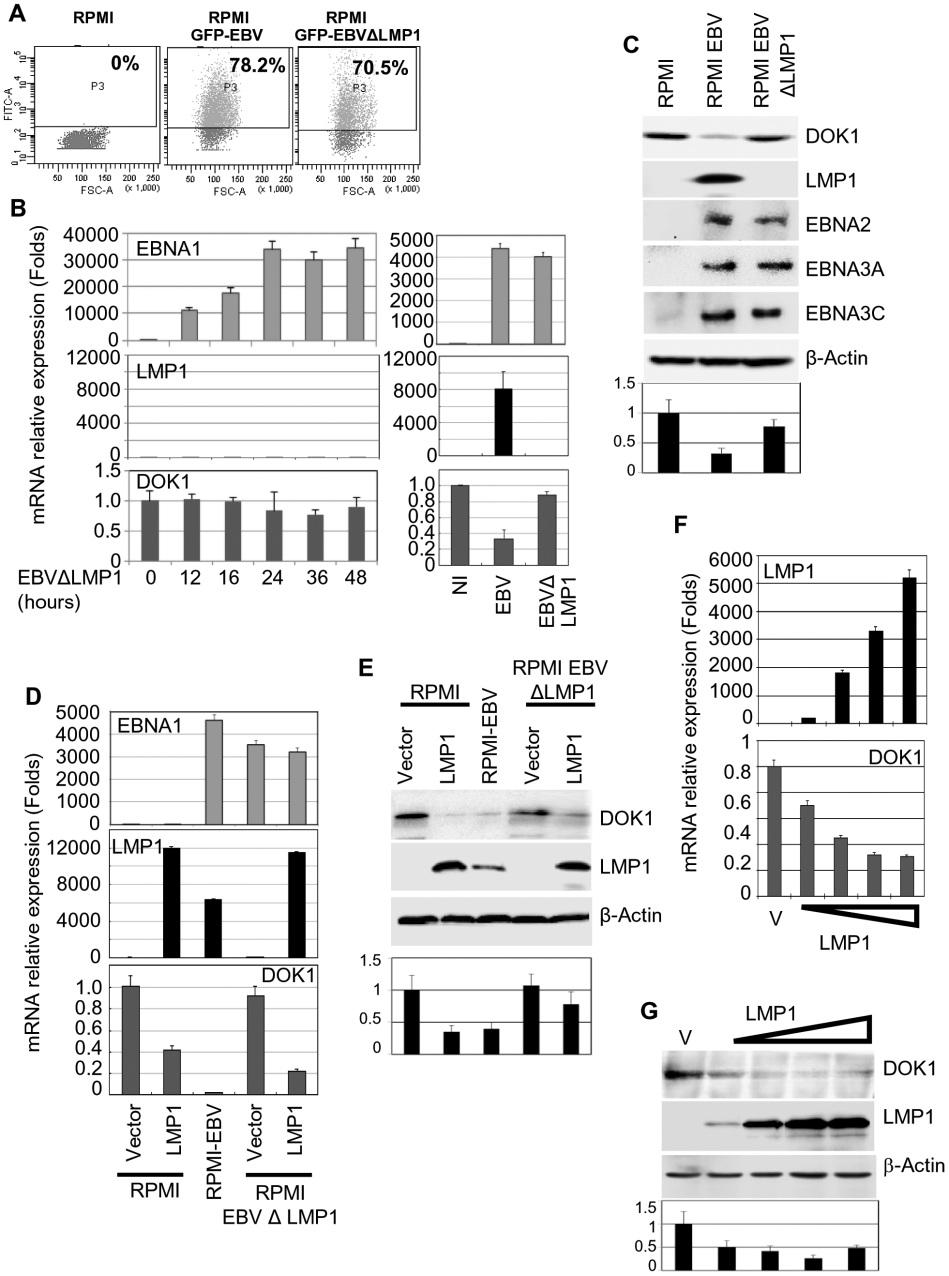
LMP1 plays a key role in EBV-mediated *DOK1* silencing. RPMI cells were infected with GFP recombinant EBV wild type (GFP-EBV) or lacking LMP1 (EBVΔLMP1). (**A**) The infection was monitored using flow cytometry for GFP expression. (**B and C**) mRNA levels of EBNA1, LMP1, *GAPDH* and *DOK1* in these cells were determined using real time PCR and the indicated proteins expression were analyzed using western blotting. Both RPMI and RPMI-EBVΔLMP1 cells were transduced using retroviral vector pLXSN empty (Vector) or expression vector pLXSN-LMP1. The cells were collected for mRNA and protein analysis. (**D and E**) The mRNA levels of EBNA1, LMP1, and *DOK1* in these cells were determined using real time PCR and normalized to *GAPDH* expression, while the indicated proteins expression were analyzed using western blot. RPMI cells were transiently transfected with increasing amounts of pcDNA3 empty plasmid (Vector) or expression vector pcDNA3-LMP1. (**F**) Cells were collected for mRNA and protein analysis. *LMP1*and *DOK1* gene expressions were measured using real time PCR for RNA levels and normalized to *GAPDH* expression. (**G**) The indicated protein levels were detected using western blotting. DOK1 protein levels were quantified from two independent immunoblots and normalized to the corresponding β-actin level (bottom of C, E and G).

The EBV oncoprotein LMP1 is essential for EBV-induced B-cell immortalization by altering cellular gene expression via the activation of several signaling pathways [28]. To determine whether LMP1 can affect the expression of *DOK1*, we infected the RPMI cells with wild-type GFP-EBV or GFP-EBV lacking the *LMP1* gene (EBVΔLMP1). The infection efficiency was monitored using flow cytometry for GFP expression (**Figure 2A**). In contrast to wild-type GFP-EBV, EBVΔLMP1 infection in primary B cells and in RPMI cells did not significantly decrease *DOK1* mRNA or protein levels (**Figure 2B and C**). Re-expression of LMP1 in EBVΔLMP1 RPMI cells by retroviral transduction restored the ability of EBV to down-regulate *DOK1* expression, while transduction of the same cells with empty retrovirus (pLXSN) did not affect *DOK1* mRNA or protein levels (**Figure 2D and E**), highlighting the key role of LMP1 in this event. Accordingly, expression of LMP1 alone in RPMI cells was sufficient to reduce *DOK1* mRNA and protein expression (**Figure 2D and E**), whereas expression of other viral proteins, such as EBNA1, 2, 3A, 3B, and 3C, did not lead to down-regulation of DOK1 protein levels (**supplementary Figure S1A–C**) In addition, transient transfection of RPMI with increasing concentrations of LMP1 expressing vector resulted in the decrease of *DOK1* expression is a dose dependent manner (**Figure 2F and G**). Together, these data underline the key role of LMP1 in EBV-mediated *DOK1* down-regulation in infected B-cells.

### LMP1 down-regulates *DOK1* expression by altering the composition of the E2F transcription complex

**Figure 3:**
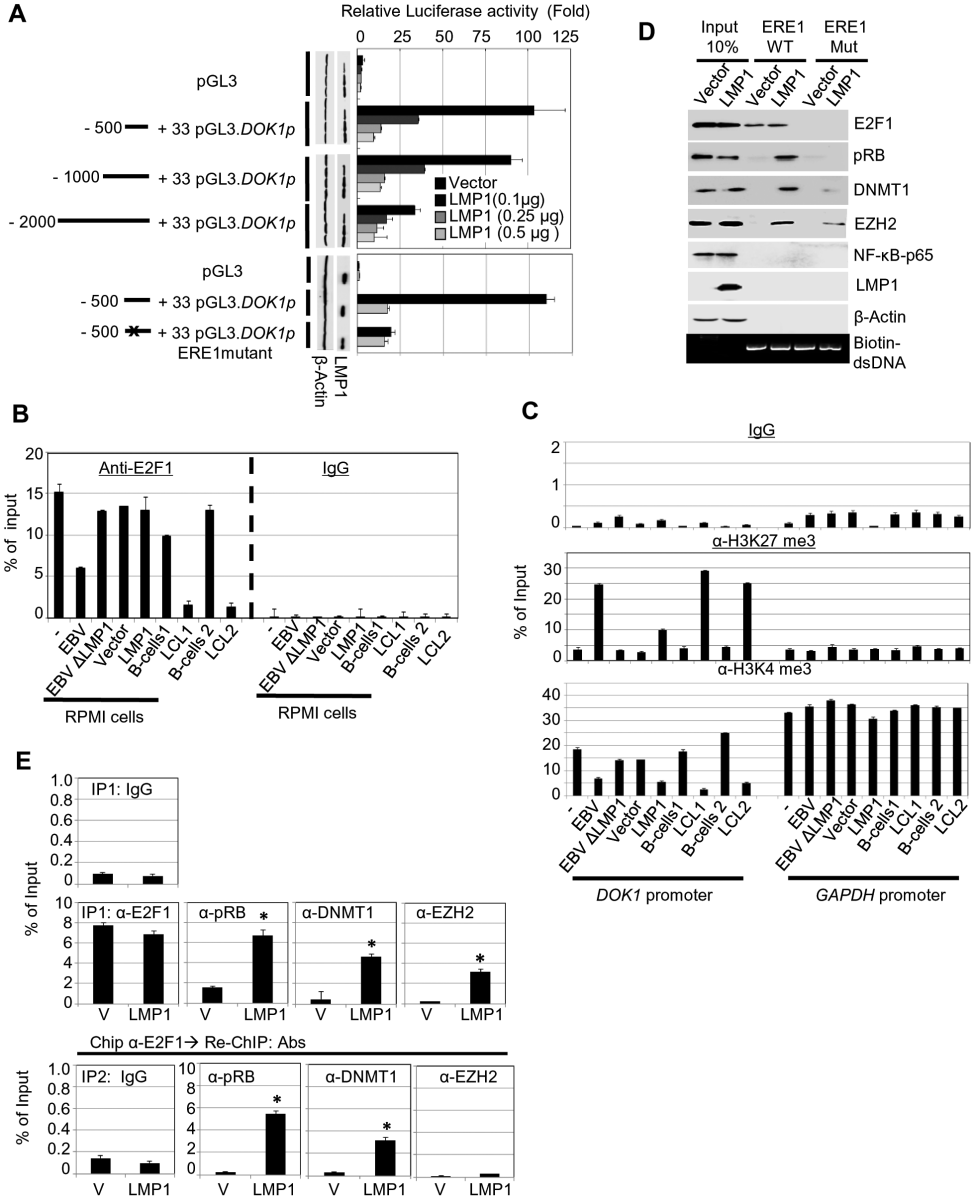
LMP1 represses *DOK1* promoter activity through the recruitment of E2F1/pRB/DNMT1 inhibitory complex. (**A**) RPMI cells were transfected with the indicated firefly luciferase reporter pGL3-*DOK1* promoter constructs along with increasing amounts of pcDNA3 LMP1. Renilla luciferase was used as an internal control for the reporter assay. After 48 hours, cells were collected and processed for luciferase activity measurement. The data are average of three independent experiments. (**B**) RPMI cells, RPMI cells infected with GFP-EBV recombinant virus, or GFP-EBVΔLMP1, RPMI cells transduced with empty pLXSN (V) or expression vector pLXSN-LMP1, and LCLs and their original primary B-cells were subjected to quantitative ChIP assay using anti-E2F1 (KH 95) antibody or IgG. The *DOK1* promoter was amplified by real-time PCR using specific primers flanking the E2F-response element located at (−498/−486). Data were calculated as percentages of enrichment of input. Error bars indicate the standard deviation from three independent experiments performed in triplicate. (**C**) The same cells from (B) were subjected to ChIP assay using the anti-H3K27 trimethylation antibody, anti-H3K4 trimethylation antibody or IgG. The *DOK1* promoter and GAPDH promoter were amplified by real-time PCR. (**D**) In vitro DNA pull-down assay. The *DOK1* promoter region containing the original E2F-response element located at (−498/−486) or a mutated one (obtained by replacing the core GGCG of the consensus sequence with AAAA), was amplified by PCR using specific 5′biotinylated primers. The PCR products (agarose gel, bottom panel) were incubated with total lysate from RPMI cells transduced with empty pLXSN (Vector) or expression vector pLXSN-LMP1, and then pulled down using streptavidin-agarose beads. Immunoblotting was used to check the recruitment of E2F1, pRB, DNMT1, EZH2 and p65 to the different PCR fragments. β-Actin was used as a negative control of binding to DNA. (**E**) Transduced RPMI cells with empty pLXSN (Vector) or expression vectorpLXSN-LMP1 were used for quantitative ChIP Re-ChIP assay. To assess the individual recruitment of E2F1, pRB, DNMT1, and EZH2 to the *DOK1* promoter, chromatin was immuno-precipitated (IP 1) with the indicated antibodies and IgG was used as a negative control (**top**). To determine the E2F1 association with the indicated factors, E2F1 chromatin complex (IP 1) was subjected to Re-ChIP (IP 2) using the indicated antibodies (**bottom**). The *DOK1* promoter was amplified using real time PCR and data from IP 1 and IP 2 were calculated as percentage of total input.

We recently showed that the E2F1 transcription factor has a key role in activation of *DOK1* transcription [29]. The 500 nucleotide upstream of the start site of the *DOK1* promoter contains three E2F1 responsive elements (RE) which appear to have a role in transcription activation; in particular the one at position −498/−486 (ERE1) [29]. Transient transfection experiments showed that LMP1 was able to efficiently inhibit the activity of −500/+33 *DOK1* promoter cloned in front of the luciferase reporter gene (**Figure 3A**). The addition of upstream regions (−1000/−500 or −2000/−500) did not modify the pattern of LMP1 inhibition (**Figure 3A**). In addition, LMP1 was not able to further decrease the activity of *DOK1* promoter harboring point mutations in ERE1 (**Figure 3A**). Together, these results suggest that LMP1 may exert its inhibitory activity targeting the regulatory complexes able to bind ERE1 within the −500/+33 region of the *DOK1* promoter. Chromatin immuno-precipitation (ChIP) experiments using an anti-E2F1 antibody showed that infection with wild-type GFP-EBV significantly decreases the recruitment of E2F1 to ERE1 in RPMI and two independent LCLs (**Figure 3B**), while EBVΔLMP1 did not have any impact on this event in RPMI (**Figure 3B**). Interestingly, LMP1 alone did not prevent the recruitment of E2F1 to the *DOK1* promoter in RPMI cells (**Figure 3B**), although it is able to efficiently down-regulate *DOK1* expression (**Figures 2D, 2E** **, and** **3A**).

We next analyzed the chromatin organization within the *DOK1* promoter in the same cells by monitoring the tri-methylation of histone H3 at lysine 4 (H3K4me3) or at lysine 27 (H3K27me3) which are events associated with transcriptionally active or inactive chromatin, respectively. According to their ability to repress *DOK1* expression, wild-type GFP-EBV or LMP1 alone induced an increase of H3K27me3 and a decrease of H3K4me3 within the *DOK*1 promoter compared with mock cells (**Figure 3C**). However, LMP1 was less efficient than the entire virus in promoting these epigenetic changes (**Figure 3C**). In summary, although LMP1 alone is not able to prevent the recruitment of E2F1 to the *DOK1* promoter, it is capable of inducing epigenetic changes and inhibition of *DOK1* transcription.

Based on these findings, we hypothesized that LMP1 mediates *DOK1* down-regulation by altering the composition of the E2F1 complex. To explore this possibility, we performed oligo pull-down experiments using biotinylated DNA probes which contain a region of the *DOK1* promoter encompassing the wild-type or mutated ERE1. Biotinylated DNA probes were incubated with protein extracts from RPMI cells transduced with empty retrovirus or with retrovirus expressing LMP1. In both extracts and as expected, E2F1 was found associated with the DNA, while only in the presence of LMP1 were three additional cellular proteins, which are usually part of negative regulatory complexes of transcription found associated with the *DOK1* promoter fragment: (i) the E2F1 inhibitor retinoblastoma (pRB), (ii) the DNA methyl-transferase DNMT1 and (iii) the polycomb-group (PcG) 2 member EZH2 (**Figure 3D**). Deletion of ERE1 prevented the association of E2F1 in both cellular extracts. In addition, in LMP1-containing extracts, mutation of ERE1 also significantly decreased the pRB and DNMT1 protein levels precipitated with DNA (**Figure 3D**), suggesting that both proteins are recruited in the same complex as E2F1. With regard to EZH2, its binding to the *DOK1* promoter was less affected by the ERE1 mutation, indicating that it is recruited by a different complex. Although LMP1 is able to activate the NF-κB pathway, no binding of the p65 transcription factor was found in both cellular extracts (**Figure 3D**). ChIP Re-ChIP experiments in mock and LMP1-expressing cells confirmed the data obtained in the pull-down assay. Indeed, Re-ChIP showed that a significant proportion of E2F1 complexes recruited to the *DOK1* promoter contains pRB and DNMT1 proteins (80% and 40% respectively), but not EZH2 (**Figure 3E**), which appears to be associated with an independent complex.

Finally, the events occurring at *DOK1* promoter were determined at early stages post-infection with EBV. We observed a significant enrichment of pRB, DNMT1 and EZH2 recruitment to *DOK1* promoter in primary naive B cells infected with recombinant GFP-EBV virus for 48 hours. Consequently, an increase of H3K27 trimethylation (∼5 folds) and CpG methylation (∼10%) was detected (**Supplementary Figure S3**). Thus, early stage of EBV infection mimics the scenario observed in LMP1-expressing cells.

In summary, these data show that LMP1 initiates the repression of *DOK1* expression by inducing the formation of transcriptional inhibitory complexes.

### LMP1-mediated NF-κB activation is required for *DOK1* down-regulation

**Figure 4:**
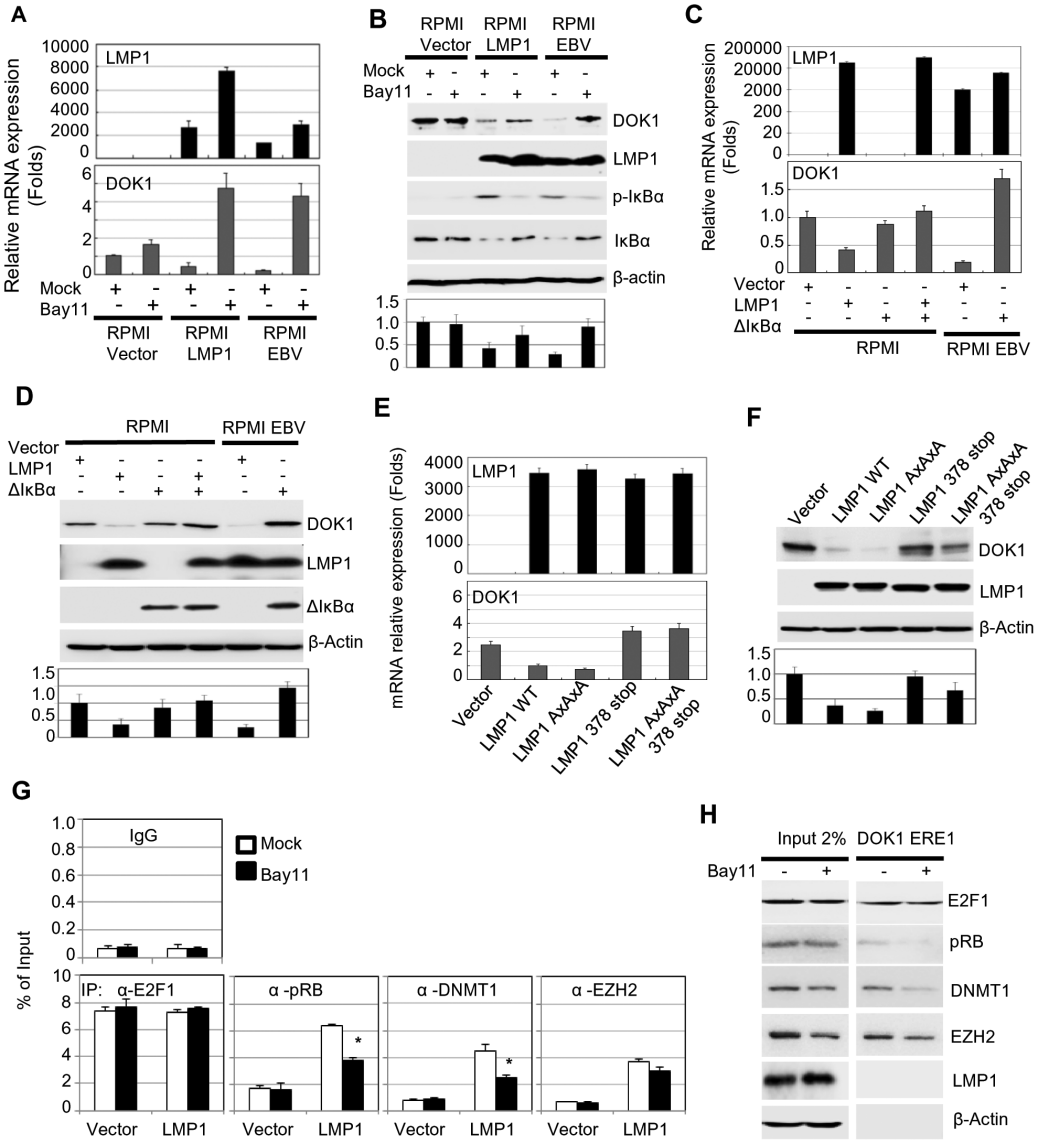
LMP1-mediated NF-κB activation is required for EBV-related *DOK1* down-regulation. RPMI cells transduced with empty retroviral pLXSN (Vector), expression vector pLXSN-LMP1, or infected with GFP-EBV recombinant virus were treated with Bay11 or the equivalent volume of DMSO (Mock). (**A**) mRNA levels of *LMP1* and *DOK1* were measured by real time PCR, and normalized to *GAPDH* expression. (**B**) The indicated proteins were detected using western blotting. RPMI cells were transfected with pcDNA3 empty plasmid (Vector), expression vector pcDNA3-LMP1 and/or expressing the super-repressor IκBα (ΔIκBα), while RPMI cells infected with GFP-EBV recombinant virus were transfected only with pcDNA3 empty (Vector) or expression vector of the super-repressor IκBα (ΔIκBα). After 48 hours, cells were collected for analysis. (**C**) mRNA levels of *LMP1* and *DOK1* were measured by real time PCR, and normalized to *GAPDH* expression. (**D**) The indicated proteins were detected using western blotting. RPMI cells were transfected with empty pLXSN (Vector), or expression vector pLXSN-LMP1 wild type (WT), LMP1 mutant for the CTAR1 domain (AxAxA), and CTAR2 domain (378 stop), or both CRAT1 and 2 domains (AxAxA/378 stop). After 48 hours, cells were harvested for expression analysis. (**E**) mRNA levels of *LMP1*, *GAPDH* and *DOK1* were measured using real time PCR. (**F**) The indicated proteins were detected using western blotting. DOK1 protein levels were quantified from two independent immunoblots and normalized to the corresponding β-actin level (bottom of B, D and F). Stable RPMI cells with empty pLXSN (Vector), or expression vector pLXSN-LMP1, were treated with Bay11 or the equivalent volume of DMSO (Mock). (**G**) Cells were subjected to quantitative ChIP assay using the indicated antibody or IgG. The *DOK1* promoter was amplified by real-time PCR using specific primers flanking the E2F-response element located at −498/−486. Data were calculated as percentages of enrichment of total input. Error bars indicate the standard deviation from two independent experiments performed in triplicate. (**H**) *In vitro* DNA pull-down assay. The *DOK1* promoter region containing ERE1 was incubated with total lysate from RPMI cells expressing LMP1 treated with Mock or Bay11, and then pulled down using streptavidin-agarose beads. Immunoblotting was used to check the recruitment of E2F1, pRB, DNMT1, EZH2. β-Actin was used as a negative control of binding to DNA.

LMP1 has the ability to activate different signaling pathways, such as NF-κB, MAPK p38, JNK, and MAPK/ERK [28]. To explore the potential role of these pathways in *DOK1* down-regulation, RPMI cells infected with recombinant GFP-EBV were treated with different chemical inhibitors specific for these signaling pathways. No change was observed in mock or GFP-EBV cells treated with the chemical inhibitors of the MAPK p38, JNK, and MAPK/ERK pathways (SB203580, S600125 and PD98059, respectively) (data not shown). However, *DOK1* mRNA and protein levels were found to be considerably increased in GFP-EBV-infected cells treated with a specific inhibitor of NF-κB (Bay11), but not in mock cells (**Figure 4A and B**). Similarly, Bay11 treatment of LMP1-expressing cells increased the *DOK1* mRNA and protein levels (**Figure 4A and B**). To further demonstrate the role of NF-κB signaling in EBV-mediated *DOK1* down-regulation, we inhibited the NF-κB canonical pathway by expressing a non-degradable deletion mutant of IκBα (Δ-IκBα) that lacks the first 36 amino acids at the N-terminus containing the IKK-phosphorylated amino acid. Similarly to Bay 11, Δ-IκBα expression in GFP-EBV RPMI cells led to an increase of transcript and protein levels of *DOK1* (**Figure 4C and D**). Accordingly, transient transfection experiments using a plasmid containing the *DOK1* promoter cloned upstream of the luciferase gene showed that Δ-IκBα antagonized LMP1 in inhibiting the *DOK1* promoter (**Supplementary Figure S2**).

The LMP1 protein has two important C-terminal cytosolic domains named C-terminal activation region 1 (CTAR-1) (residues 194–232) and 2 (CTAR-2) (residues 351–386). Both the CTAR1 and CTAR2 domains have the ability to activate the NF-κB pathway through their interactions with tumor necrosis factor receptor (TNFR)-associated factors (TRAFs) [30], and TNFR-associated death domain protein (TRADD) [31], respectively. In particular, the CTAR2 domain is required for the activation of the canonical NF-κB pathway, while the CTAR1 domain is critical for the stimulation of the non-canonical NF-κB pathway [32]. The LMP1 mutants AxAxA (mutated CTAR1), 378 stop (deleted in CTAR2) and AxAxA/378 stop (mutated CTAR1 and deleted CTAR2) were expressed in RPMI cells. Both LMP1 378 stop and AxAxA/378 stop mutants failed to down-regulate the *DOK1* gene, but not the LMP1 AxAxA mutant, which still retained its ability to suppress *DOK1* expression at similar levels of wild-type LMP1 (**Figure 4E and F**). Therefore, LMP1 down-regulates *DOK1* expression through its CTAR2 domain. In addition, we investigated whether the LMP1-mediated NF-κB activation plays a role in the formation of inhibitory complexes and their recruitment to the *DOK1* promoter. LMP1-expressing RPMI cells were cultured in the presence of NF-κB inhibitor Bay11. No significant change in pRB and DMNT1 intracellular levels was observed, whereas EZH2 levels were slightly decreased (**Figure 4H**). However, oligo pull-down experiments clearly showed that inhibition of the NF-κB signaling affected the binding efficiency of pRB and DNMT1 to *DOK1* promoter, while E2F1 and EZH2 continued to be associated with the DNA (**Figure 4H**). ChIP assay confirmed that inhibition of NF-κB significantly decreased the recruitment of pRB and DNMT1 to the *DOK1* promoter (**Figure 4G**). Together, the data show that activation of the canonical NF-κB pathway by LMP1 is an important event for the down-regulation of *DOK1* expression.

### *DOK1* gene silencing through DNA methylation is associated with EBV infection in B-cells

**Figure 5:**
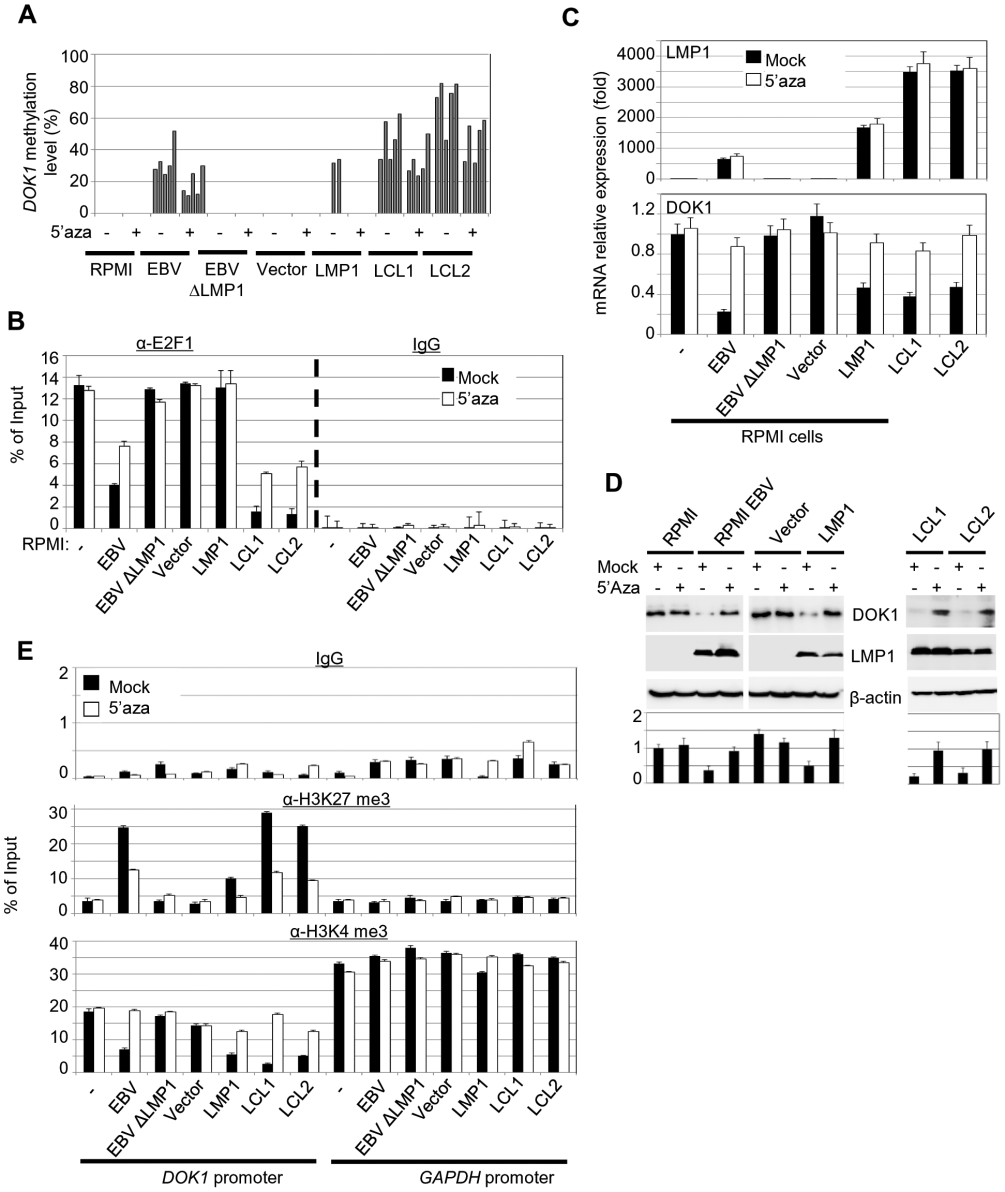
5-Aza treatment rescue *DOK1* expression in EBV infected cells. Cells were treated with 1 µM methyl-transferase inhibitor 5-Aza-2′deoxycytidine (5-Aza) for 4 days or equivalent volume of DMSO (Mock), then collected for analysis. (**A**) DNA methylation levels of the *DOK1* promoter were measured using pyrosequencing. Each bar represents the percentage of methylation for individual CpG sites. (**B**) Quantitative ChIP assay using anti-E2F1 (KH 95) antibody or IgG. The *DOK1* promoter was amplified by real-time PCR using specific primers flanking the E2F-response element located at (−498/−486). Data were calculated as percentages of enrichment of input. Error bars indicate the standard deviation (SD) from two independent experiments performed in triplicate. (**C**) The mRNA expression levels of *LMP1*, *GAPDH* and *DOK1* were determined using real time PCR. (**D**) The indicated proteins were analyzed using western blotting. DOK1 protein levels were quantified from two independent immunoblots and normalized to the corresponding β-actin level (bottom). (**E**) ChIP assays were carried out using anti-H3K27 trimethylation antibody, anti-H3K4 trimethylation antibody or IgG. The *DOK1* promoter and *GAPDH* promoter were amplified by real-time PCR. Data were calculated as percentages of enrichment of input.

In our previous study [9], we reported that *DOK1* expression is repressed in 64% of Burkitt's lymphoma cell lines through DNA hypermethylation of its promoter. These findings prompted us to assess whether hypermethylation of the *DOK1* promoter could be ascribed to the presence of EBV. Using pyrosequencing and real-time PCR, respectively, *DOK1* methylation and expression levels were measured in our experimental model. EBV infection of RPMI cells led to hypermethylation of *DOK1* promoter (**Figure 5A**). This phenomenon was even more evident in LCLs (**Figure 5A**). EBVΔLMP1 was unable to promote *DOK1* promoter methylation, further underlining the importance of the viral oncoprotein in this event. However, in agreement with the fact that LMP1 alone is unable to displace E2F1 from the *DOK1* promoter, low DNA methylation was detected in RPMI cells expressing LMP1 alone (**Figure 5A**). In addition, no further methylation was observed in RPMI cells co-expressing LMP1 with other EBV proteins, i.e. EBNA3A, 3B, or 3C (data not shown), suggesting that a more complex pattern of viral gene expression is required to induce hypermethylation of *DOK1* promoter. Treatments with the methyl-transferase inhibitor 5-Aza-2′-deoxycytidine (5-Aza) significantly affected *DOK1* promoter methylation in LCLs and RPMI cells infected with EBV (**Figure 5A**). As expected, 5-Aza treatment rescued the recruitment of E2F1 to the *DOK1* promoter in GFP-EBV infected cells, while no change was observed in LMP1-expressing cells (**Figure 5B**). However, an increase of *DOK1* mRNA and protein levels was observed upon exposure to 5-Aza in GFP-EBV-infected cells (RPMI or LCLs) as well as in LMP1-expressing RPMI cells (**Figure 5C and D**). This event correlates with the decrease of H3K27me3 and the increase of H3K4me3 levels (**Figure 5E**).

Together, these data show that EBV induces hypermethylation of the *DOK1* promoter. Although expression of LMP1 alone marginally promotes DNA methylation, deletion of its gene in the EBV genome prevents the occurrence of this event. Thus, LMP1 appears to be essential, but not sufficient for hypermethylation of the *DOK1* promoter.

### Re-expression of DOK1 in LCLs decreased cellular proliferation and induced apoptosis

**Figure 6:**
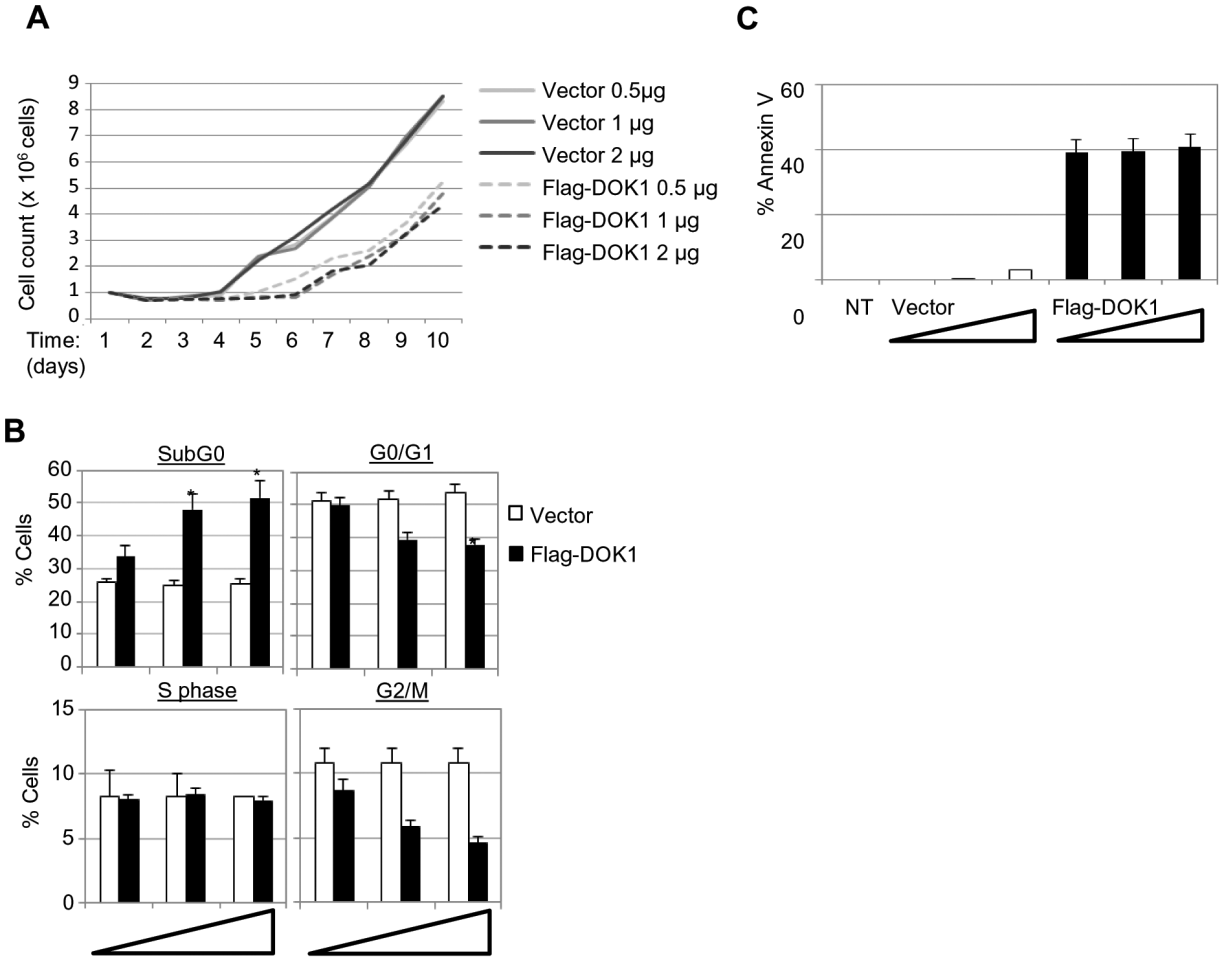
(A) LCL cells were transfected with the indicated amounts of empty pcDNA3 (Vector) or expression vector pcDNA3-Flag-DOK1. After 24(**B**) LCL cells were monitored for cell cycle analysis 48 hours after being transfected with the indicated amounts of pcDNA3 empty (Vector) or expression vector pcDNA3-Flag-DOK1. Cells in different cycle phases (SubG0, G0/G1, S, or G2/M) are represented as percentage of total cells. (**C**) The same cells from (B) were monitored for apoptosis using Annexin V staining. Non transfected cells were used as control (NT). Error bars indicate the SD from two independent experiments. Data were analyzed using Student's t test (*, P<0.05).

To understand the biological significance of EBV-induced *DOK1*-down-regulation, we re-expressed DOK1 in LCLs. We observed that ectopic DOK1 levels decreased LCL proliferation in a dose-dependent manner (**Figure 6A**). Consistently with these observations, DOK1 induced a significance decrease of cell populations in G0/G1 and G2/M phases (**Figure 6B**). In addition, high levels of DOK1 led to a significant increase of subG0 population and AnnexinV-positive cells (**Figure 6B and C**).Together, these data demonstrate the role of DOK1 in inhibiting cell proliferation induced by EBV and promoting both cell growth arrest and apoptosis.

## Discussion

Several studies have demonstrated that the loss of DOK1 function is a key event in human carcinogenesis [1], [3], [4], [9], [33]. Indeed several mechanisms of *DOK1* inactivation have been characterized so far *DOK1* expression was found to be silenced by hypermethylation of its promoter in a variety of human cancers, including, head and neck, lung, gastric and liver cancer as well as in Burkitt's lymphoma-derived cell lines [9], [10], [11]. In addition, *DOK1* was found to be mutated in chronic lymphocytic leukemia (CLL) [8]. At the protein level, DOK1 is targeted for proteasome degradation triggered by oncoprotein kinases (OTKs) such as p210^bcr-abl^ and oncogenic forms of Src [7].

A recent study has provided evidence that *DOK1* inactivation also occurs in virus-induced cancers [10]. Indeed, a correlation between *DOK1* aberrant hypermethylation and the presence of hepatitis B virus (HBV) has been reported in hepatocellular carcinoma (HCC) [10]. Similarly, the expression of *DOK1* mRNA was found to be down-regulated in cell lines derived from Burkitt's lymphoma [34], a pathological condition associated with EBV infection. However, these initial findings do not provide evidence about whether the down-regulation of *DOK1* expression is directly induced by the viral proteins or is a consequence of the chromosomal alterations occurring during the carcinogenic processes. In this study, we demonstrate for the first time that EBV is directly involved in the inhibition of *DOK1* expression. Our data show that the EBV LMP1 oncoprotein plays a key role in this event. Indeed, an EBV mutant lacking the entire *LMP1* gene was unable to inhibit *DOK1* transcription, while re-expression of LMP1 in cells infected with the EBVΔLMP1 mutant fully restored the ability of EBV to decrease *DOK1* mRNA and protein levels. Expression of LMP1 alone in human cancer B-cells was sufficient to efficiently inhibit *DOK1* transcription by promoting the formation of a transcriptional repressor complex containing E2F1, pRB, and the DNA methyl-transferase DNMT1. In addition, deletion of the E2F1-binding element (ERE1) strongly affected the binding of three cellular proteins to the *DOK1* promoter, and a Re-ChIP assay confirmed that E2F1 is the carrier of pRB and DNMT1. We also observed that LMP1 promotes the recruitment of the histone-lysine N-methyl-transferase EZH2 independently of E2F1, leading to an increase in the level of H3K27me3. In agreement with the recruitment of the two epigenetic enzymes, an increase in H3K27me3 and DNA methylation levels was detected at the *DOK1* promoter.

It has previously been shown that LMP1 is able to increase the expression and activity of DNA methyl-transferases (DNMT 1, 3a, and 3b), which could explain the increase of the *DOK1* promoter methylation. Interestingly, DNA methylation was strongly enhanced in B-cells infected by the entire virus compared with cells expressing only LMP1. Thus, it is likely that additional viral products may cooperate with LMP1 in promoting *DOK1* silencing via DNA methylation. No down-regulation of *DOK1* was observed when EBNA1, 2, 3A, 3B, and 3C are expressed in RPMI cells. In addition, none of these viral proteins further stimulate DNA methylation at *DOK1* promoter when co-expressed with LMP1 **(data not shown)**. Thus, a more complex pattern of viral gene expression may be involved in the hyper-methylation of *DOK1* promoter. Most importantly, we show that in EBV-infected B-cells the DNA methylation extends over a large region of the *DOK1* promoter including ERE1 that loses the ability to recruit the active form of E2F1. Inhibition of DNA methylation significantly increases *DOK1* transcription in LMP1-expressing cells as well as EBV-infected cells.

**Figure 7:**
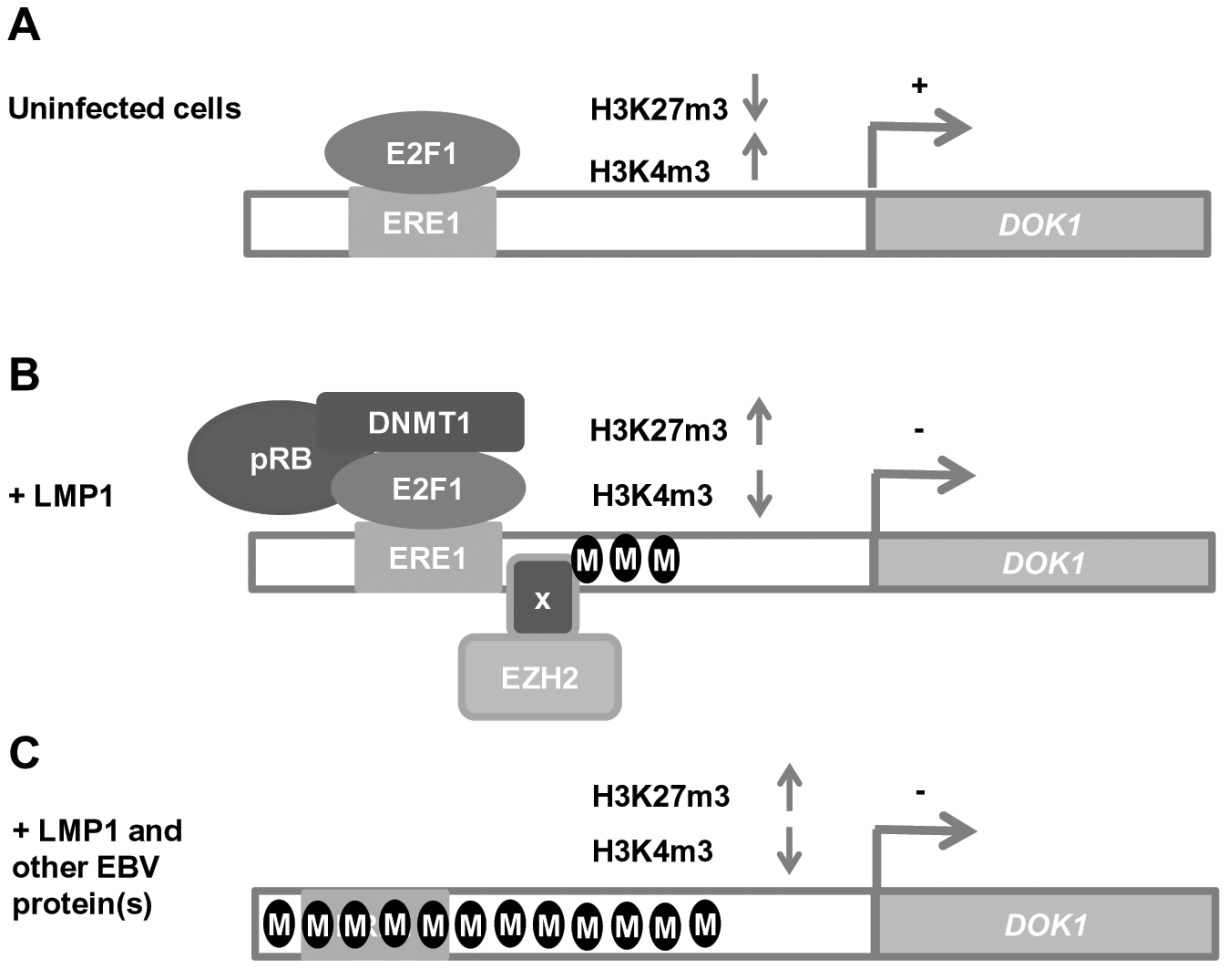
Schematic model of *DOK1* gene regulation in EBV-infected cells. (**A**) In uninfected cells, *DOK1* expression is activated via the recruitment of the active form of the E2F1 transcription factor to its response element located at (−498/−486) on the *DOK1* promoter. (**B**) In cells expressing the oncoprotein LMP1, *DOK1* is down-regulated through the recruitment of the inhibitory complexes E2F1/pRB/DNMT1 and EZH2 to its promoter region. These complexes lead to the induction of partial DNA methylation and the increase of H3K27 trimethylation levels, respectively. (**C**) In EBV-infected cells, *DOK1* is repressed through heavy DNA methylation of its promoter region and the increase in H3K27 trimethylation level. These events likely induce conformational changes in the chromatin, which become less permissive to E2F1 transcription factor recruitment.

In summary, based on our findings, a two-step model can be proposed for EBV in the inhibition of *DOK1* expression (**Figure 7**). In the first step, LMP1 favors the formation and recruitment of transcriptional repressor complexes containing E2F1/pRB/DNMT1 and EZH2. These complexes induce epigenetic changes in the *DOK1* promoter region, leading to its inhibition. In the second step, LMP1 in collaboration with other EBV proteins leads to further increase of DNA methylation which in turn results in a loss of all transcriptional regulatory complexes and a strong repression of the *DOK1* promoter. These data corroborate our previous studies that highlighted the key role of E2F1 and DNA methylation in the regulation of *DOK1* expression [29]. Our data also show that the LMP1-induced *DOK1* down-regulation is linked to activation of the NF-κB canonical pathway. Indeed, NF-κB activation by LMP1 plays a role in the formation and recruitment of inhibitory complex E2F1/pRB/DNMT1 to the *DOK1* promoter. Although we did not observe any recruitment of p65 to the *DOK1* promoter, neither by DNA-pull-down assay nor by chromatin immuno-precipitation (data not shown), we cannot exclude the involvement of other NF-κB transcription factors.

Until now, several studies reported that DNA methylation patterns were higher in EBV positive tumors compared to the EBV-negative ones and that EBV infection was clearly demonstrated to induce specific methylation epigenotypes that lead to silencing of multiple tumor suppressor genes such as *BIM*, *p16^INK4A^*, *p14^ARF^*, *p73*, *E-cadherin* and *PTEN* in EBV–associated nasopharyngeal and gastric cancers [17], [35], [36], [37], [38]. While these events are believed to be caused by elevated levels of DNMTs induced by LMP1 and 2, the mechanisms establishing the methylation patterns themselves are unknown. As DNA methyl-transferases have little specificity *in vitro*, we propose the notion that LMP1 triggers *DOK1* gene repression through the recruitment of DNMT1 to its promoter in a specific manner via E2F1-binding to its response element, and this event might be an early step for EBV-induced DNA methylation. As some of the genes listed above are targets of E2F1 [39], [40], it will be interesting to see whether their methylation patterns are also specific to the recruitment of the inhibitory complex E2F1/pRB/DNMT1. Moreover, EBV appears to have an initiator role of epigenetic alterations and therefore inducing oncogenesis, however, the latency expression patterns of EBV genes differ in different cancers, which make unclear the contribution of the virus to some types. One explanation would be that EBV-induced epigenetic changes, such as EBV-mediated DNA methylation of *DOK1* promoter, are stable events and could also persist even after the changes in EBV latent gene expression. As *DOK1* gene silencing was found to be related to its promoter hypermethylation in gastric cancer [11], it will be important to investigate whether these events are associated with the presence of EBV in these cancers and others.

In conclusion, the present study sheds light on the association between EBV infection and *DOK1* down-regulation in B-cells. It provides novel insights into the regulation of *DOK1* in viral-related carcinogenesis, and could define it as a potential cancer biomarker and an attractive target for epigenetic-based therapy.

## Materials and Methods

### Expression vectors

Cellular and viral genes were expressed using the retroviral vector pLXSN (Clontech, Palo Alto, CA) or the expression vector pcDNA-3 (Invitrogen). The pLXSN-LMP-1 and the mutants LMP-1AxAxA, LMP-1 378 stop, and LMP-1AxAxA/378 stop constructs have been previously described [41]. The pGL3 basic luciferase reporter (Promega) and pGL3 containing the *DOK1* promoter constructs have been described previously [29], The NF-κB super-repressor Δ-IκBα, which lacks the coding sequence of the first 36 N-terminal amino-acids, was kindly provided by Dr Elliot Kieff (Harvard Medical School, Boston, Massachusetts, USA). The expression plasmids pDEST-myc-EBNA1, pSG5-EBNA2, pDEST-myc-EBN3A1, pDEST-myc-EBNA3B, pDEST-myc-EBNA3C were kindly provided by Dr Evelyne Manet (ENS, Lyon, France).

### Cells, transfection, and chemicals

RPMI 8226 cells were kindly provided by Dr Christophe Caux (Centre Léon Bérard, Lyon, France). The EBV-negative immortalized B-cells, BJAB were previously described [42], and the Louckes cells were kindly provided by Dr Evelyne Manet (ENS, Lyon, France). The primary B-cells were isolated from total blood of healthy donors using negative selection EasySep or RosetteSep (StemCell Technologies). Primary naive B cells and RPMI cells were infected with recombinant GFP-EBV, and GFP-EBVΔLMP-1 as described in [43], [44], [45], RPMI pLXSN-empty or pLXSN-LMP1 cell lines were generated as described previously [41]. Expression of LMP-1 wild-type, LMP-1 AxAxA , LMP1 378 stop, and LMP1 AxAxA/378 stop mutants in RPMI was achieved by transduction with recombinant retroviruses [41]. The EBV-immortalized lymphoblastoid cell lines (LCLs) were generated in this study by infecting primary B-cells isolated from different donors with recombinant EBV expressing GFP, as described previously [41]. Primary and immortalized B-cells were cultured in RPMI 1640 medium (GIBCO, Invitrogen life Technologies, Cergy-Pontoise, France) supplemented with 10% FBS, 100 U/ml penicillin G, 100 mg/ml streptomycin, 2 mM L-glutamine, and 1 mM sodium pyruvate (PAA, Pasching, Austria). Expression plasmids were transiently transfected in cells using Xtreme gene 9 reagents (Roche) according to the manufacturer's protocol.

For treatment, cells were incubated in media containing different reagents: with a final concentration of 1 µM of the NF-κB pathway inhibitor Bay11 in dimethyl sulfoxide (DMSO) for 6 hours. Inhibition of DNA methylation was performed by incubation for 4 days with 5-aza-2′-deoxycytidine (5-aza) at 1 µM (Sigma) dissolved in DMSO. Cells were then harvested for analysis.

### Quantitative RT-PCR

Total RNA was extracted using TRIzol reagent (Life Technologies). Reverse transcription was performed using the RevertAid H Minus First Strand cDNA synthesis kit (Fermentas) according to the manufacturer's protocol. Real-time PCR was performed using the following gene-specific primers:

*DOK1*: Fw ATGGACGGAGCAGTGATGGA, Rev CCCAGGTCTTCCTCCACCTC

*LMP1*: Fw CCCCCTCTCCTCTTCCATAG, Rev GCCAAAGATGAACAGCACAA

*EBNA1*: Fw GGACCCGGCCCACAACCTG, Rev CTCCTGCCCTTCCTCACCCTCATC

*GAPDH*: Fw GAAGGTGAAGGTCGGAGTC, Rev AAGATGGTGATGGGATTTC. Data were analyzed using the ΔΔ^CT^ method.

### Antibodies and immunoblotting

The following antibodies were used: anti-DOK1 (ab8112, Abcam), anti-E2F1 (KH-95; Santa Cruz Biotechnology), anti- β-Actin C4 (MP Biomedicals), anti-LMP1 (S12), anti- phosphor IκBα (#9246, Cell Signaling Technology), anti-total IκBα (#9242, Cell Signaling Technology), mouse IgG, rabbit IgG (Santa Cruz Biotechnology), anti-p65 (#3034, Cell Signaling Technology), anti-H3K4me3, and anti-H3K27me3 (Epigentek), anti-EZH2 (AC22; Cell Signaling Technology), anti-pRB (4H1, Cell Signaling Technology), anti-DNMT1 (60B1220, Abnova), anti-EBNA1 (1EB12, Santa Cruz Biotechnology), anti-EBNA2 (Novocastra), anti-EBNA3A (Exalpha), anti-EBNA3C (ab16128, Abcam). Immunoblotting was performed as described previously [29].

### Reporter assays

Cells were transfected with 0.250 µg of pGL3 or *DOK1* promoter constructs along with other experimental plasmids using X-tremeGENE 9 (Roche Diagnostics). The Renilla construct was included for normalization of transfection efficiency. At 48 hours after transfection, cells were harvested and the enzyme activities of firefly and Renilla luciferases were measured using the Dual-Luciferase reporter assay system (Promega). The luminescence signal was quantified using an Optocomp I luminometer (MGM Instruments). Each condition was used in triplicate and replicated in different independent experiments.

### Chromatin immuno-precipitation (ChIP)

For each reaction, 10^6^ cells were cross-linked with 1% formaldehyde, harvested and subjected to sonication to shear the chromatin into fragments of 0.2 kb, immuno-precipitated with 2 µg of appropriate antibody, and then processed according to the standard protocol for ChIP analysis from Cell Signaling Technology.

Low cell ChIP kit (Diagenode) was used for primary B cells and infected with EBV for 48 hours. 50 000 cells per reaction were processed according to the manufacturer's protocol.

The input and immuno-precipitated DNA from both methods (standard and low cell) were then analyzed by real-time PCR using primers flanking the E2F-response element (−498/−486) of the *DOK1* promoter: Fw GCCAAAACCGAGGACTTTCG, Rev CATCACTGCTCCGTCCATGG, or primers for *GAPDH* promoter: Fw GACGGCCGCATCTTCTTGT, Rev CCTGGTGACCAGGCGC. Data were calculated as a percentage of enrichment of input.

### Re-ChIP assay

Following the initial anti-E2F1 ChIP (performed as above using 10^7^ cells and 10 µg of anti E2F1 KH-95 antibody), up to the final wash step with TE buffer, E2F1–chromatin complexes were eluted by the addition of 10 mM dithiothreitol (DTT) and incubated for 30 minutes at 37°C. Supernatants were diluted 1∶20 with re-ChIP buffer (1% Triton X-100; 20 mM Tris–HCl, pH 8.1; 2 mM EDTA; 150 mM NaCl; supplemented with protease inhibitors), and immuno-precipitated a second time (IP 2) using 4 µg of antibody against pRB, DNMT1, and EZH2. IgG was used as negative control. The Re-ChIP mixtures were incubated overnight at 4°C with rotation. Isolation and purification of associated DNA were carried out as described for the standard ChIP experiment. The binding of each factor was determined by real-time PCR as previously described. Data were calculated as a percentage of enrichment of total input.

### DNA pull-down assay

Cells were lysed by sonication in HKMG buffer (10 mM HEPES, pH 7.9; 100 mM KCl; 5 mM MgCl2; 10% glycerol; 1 mM dithiothreitol (DTT); and 0.5% NP-40) containing protease and phosphatase inhibitors. Cellular debris was removed by centrifugation. Then, 1 mg of total lysate was pre-cleared with 40 µl of streptavidin-agarose beads (Thermo Scientific) for 1 hour at 4°C, with rotation, and incubated with 2 µg of biotinylated PCR product oligonucleotides and 20 µg of poly (dI-dC) for 16 hours at 4°C, with rotation. Biotin-oligonucleotide-protein complexes were collected with 60 µl of streptavidin-agarose beads for 1 hour at 4°C, with rotation, washed twice with HKMG buffer, separated on SDS-PAGE, and detected by western blotting. The biotinylated double-stranded oligonucleotides were amplified using the same primers as for ChIP with 5′ biotin.

### DNA extraction and pyrosequencing

Genomic DNA was extracted using the QIAamp DNA minikit (Qiagen) and bisulfite converted using the EZ DNA Methylation-Gold kit (Zymo Research). Converted DNA was then subjected to Pyrosequencing (Qiagen) as previously described [46]. The primers used to measure the methylation of *DOK1* promoter were: Fw GAGGTGGAGGAAGATTTG, Rev BIOTIN-CCACACTCACACACTCAA, and sequencing primer AGTTTTGGGGGTGGT. The percentage of methylation was evaluated as the mean of each CpG analyzed.

### Flow cytometry analysis

To determine cell cycle profile, cells were collected 48 hours post-transfection with empty pCDNA3 (Vector) or expression vector pCDNA3-Flag-DOK1, washed twice with PBS 1×, and then cell pellets were re-suspended in 70% ethanol while vortexing, in order to prevent cell clumps. After ethanol fixation (30 minutes at 4°C) the cells were rewashed in PBS 1× and finally re-suspended in PBS 1×+ 100 µg/mL RNAse (Roche) + 25 µg/mL of Propidium iodide (Sigma).

Apoptotic cells were detected using the PE Annexin V apoptosis detection kit I (BD Pharmingen) according to the manufacturer's instructions.

Stained cells for cell cycle and for apoptosis were detected using the BD FACSCanto II flow cytometer (BD Biosciences) and analyzed using FACSDiva software.

### Ethics statement

Blood samples from healthy donors were provided by the Etablissement Français du Sang (EFS, Lyon, France) after being anonymized. All participants signed a written informed consent.

## Supporting Information

Figure S1 **Expression of latent EBV proteins EBNA1, 2, 3A, 3B, and 3C failed to down-regulate** ***DOK1*** **gene expression.** RPMI cells were transfected with 0.5 µg of empty vector or expression vector of myc-EBNA1 (**A**), EBNA2 (**B**), myc-EBNA3A, 3B, or 3C (**C**). After 48 hours post-transfection, the expression of the indicated proteins was determined using western blotting.
(TIF)

Figure S2 **Inhibition of LMP1 mediated NF-κB activation leads to the rescue of** ***DOK1*** **promoter activity and protein expression.** (**A**) RPMI cells were transfected with pGL3 basic vector, or containing the *DOK1* promoter construct (−500/+33) along with pcDNA3 empty (Vector), expressing LMP1 or different amounts of the super-repressor IκBα (ΔIκBα). The Renilla luciferase was used as an internal control for the reporter assay. After 48 hours, the cells were harvested and the luciferase activities were measured. (**B**) The expression of the indicated proteins was determined using western blotting.
(TIF)

Figure S3 **Early stage infection with EBV leads to epigenetic repression of** ***DOK1*** **expression in primary B cells.** (**A**) Primary B cells were isolated from healthy donor blood using negative selection, and then infected with GFP-EBV recombinant virus. Genomic DNA was extracted at different time points 12, 16, 24, 36, and 48 hours post infection, and DNA methylation of *DOK1* promoter was measured using pyrosequencing. (**B**) Primary B cells were infected with GFP-EBV recombinant virus for 48 hours. Quantitative low cell ChIP assay was performed to measure the individual recruitment of E2F1, pRB, DNMT1, and EZH2 to the *DOK1* promoter, and the levels of histone 3 modifications (H3K27 trimethylation or H3K4 trimethylation). Non infected primary B cells were used as control. Data was calculated as percentage of enrichment of total Input. Statistical significance was measured using Student's t test (*, p value<0.05).
(TIF)
