# Supplementary figures and images for: Epstein-Barr Virus Down-Regulates Tumor Suppressor DOK1 Expression
Source: PLoS Pathog. 2014 May 8;10(5):e1004125. doi: 10.1371/journal.ppat.1004125 (PMC4014463; doi:10.1371/journal.ppat.1004125)

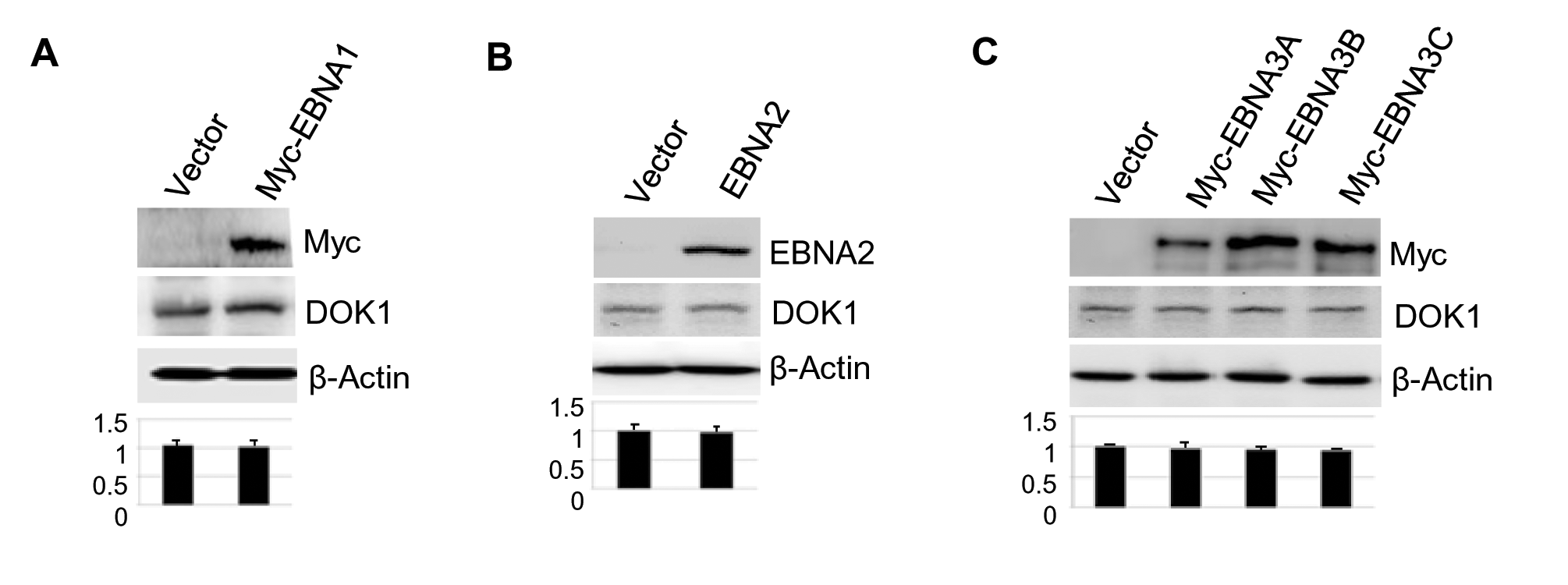

Supplement: Figure S1 — Expression of latent EBV proteins EBNA1, 2, 3A, 3B, and 3C failed to down-regulate DOK1 gene expression. RPMI cells were transfected with 0.5 µg of empty vector or expression vector of myc-EBNA1 (A), EBNA2 (B), myc-EBNA3A, 3B, or 3C (C). After 48 hours post-transfection, the expression of the indicated proteins was determined using western blotting. (TIF) [file ppat.1004125.s001.tif]

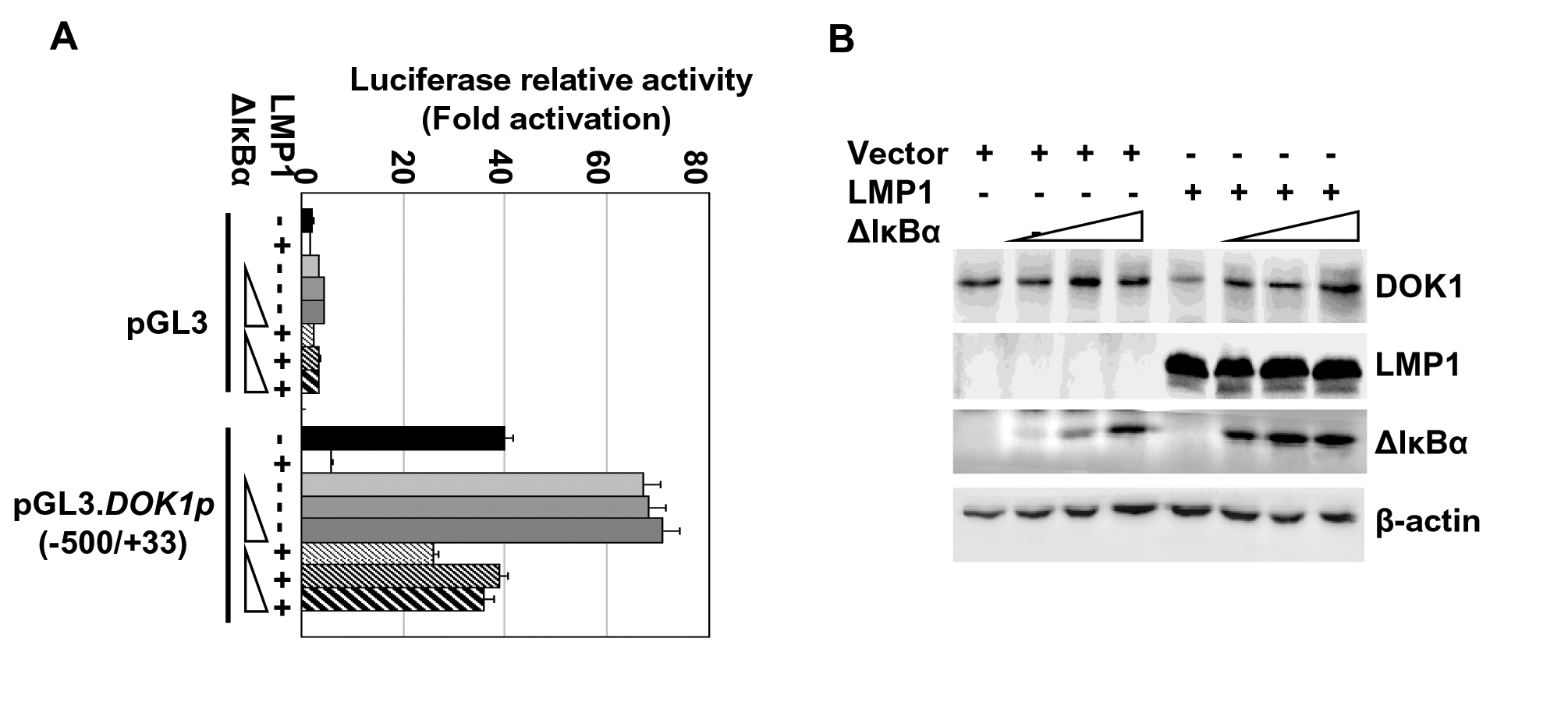

Supplement: Figure S2 — Inhibition of LMP1 mediated NF-κB activation leads to the rescue of DOK1 promoter activity and protein expression. (A) RPMI cells were transfected with pGL3 basic vector, or containing the DOK1 promoter construct (−500/+33) along with pcDNA3 empty (Vector), expressing LMP1 or different amounts of the super-repressor IκBα (ΔIκBα). The Renilla luciferase was used as an internal control for the reporter assay. After 48 hours, the cells were harvested and the luciferase activities were measured. (B) The expression of the indicated proteins was determined using western blotting. (TIF) [file ppat.1004125.s002.tif]

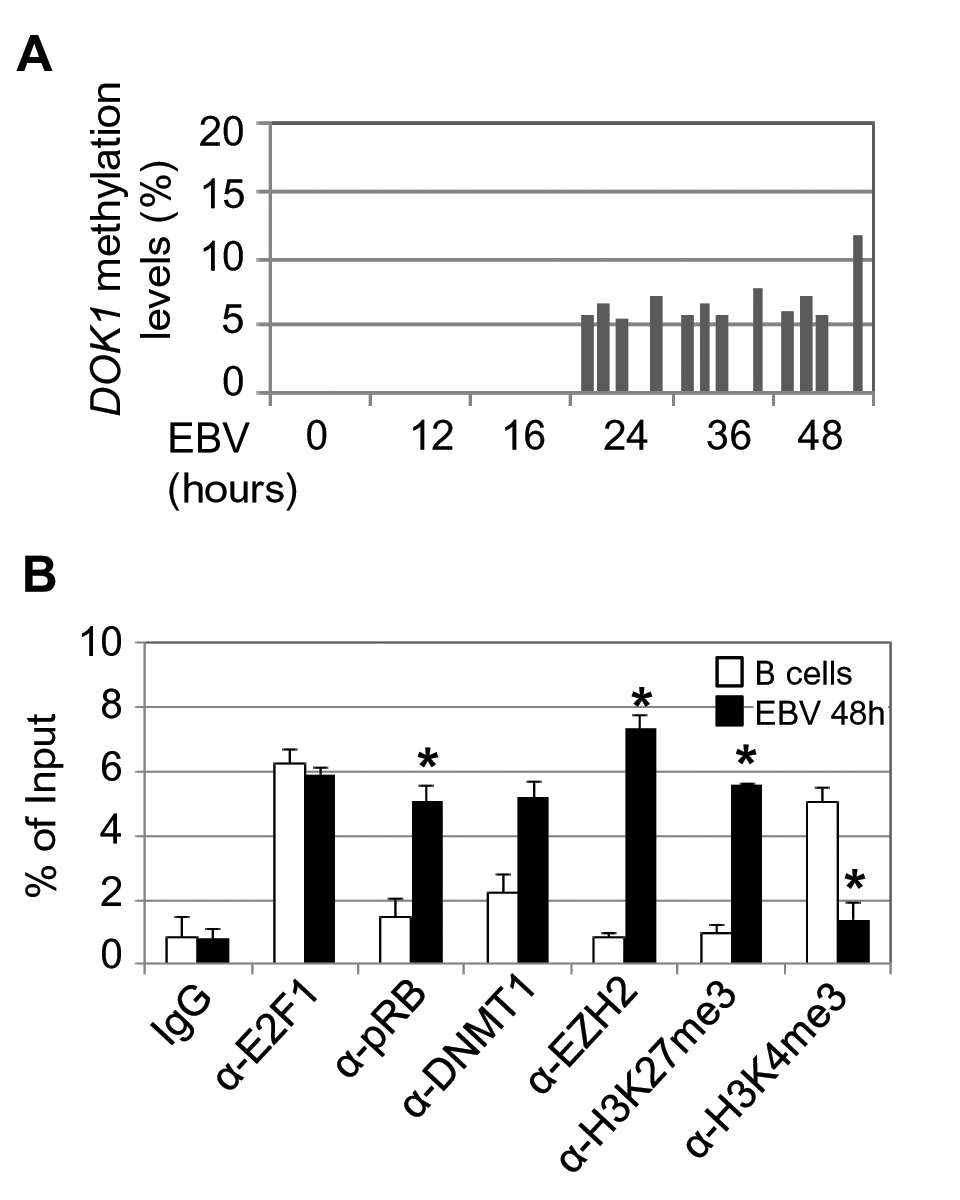

Supplement: Figure S3 — Early stage infection with EBV leads to epigenetic repression of DOK1 expression in primary B cells. (A) Primary B cells were isolated from healthy donor blood using negative selection, and then infected with GFP-EBV recombinant virus. Genomic DNA was extracted at different time points 12, 16, 24, 36, and 48 hours post infection, and DNA methylation of DOK1 promoter was measured using pyrosequencing. (B) Primary B cells were infected with GFP-EBV recombinant virus for 48 hours. Quantitative low cell ChIP assay was performed to measure the individual recruitment of E2F1, pRB, DNMT1, and EZH2 to the DOK1 promoter, and the levels of histone 3 modifications (H3K27 trimethylation or H3K4 trimethylation). Non infected primary B cells were used as control. Data was calculated as percentage of enrichment of total Input. Statistical significance was measured using Student's t test (*, p value<0.05). (TIF) [file ppat.1004125.s003.tif]
